# Generation and Characterization of the Anp32e-Deficient Mouse

**DOI:** 10.1371/journal.pone.0013597

**Published:** 2010-10-26

**Authors:** Patrick T. Reilly, Samia Afzal, Andrew Wakeham, Jillian Haight, Annick You-Ten, Kathrin Zaugg, Joanna Dembowy, Ashley Young, Tak W. Mak

**Affiliations:** 1 Department of Cellular and Molecular Research, National Cancer Centre Singapore, Singapore, Singapore; 2 Campbell Family Cancer Research Institute, University Health Network, Toronto, Ontario, Canada; 3 Graduate Program in Immunology, University of Toronto, Toronto, Ontario, Canada; University of Florida, United States of America

## Abstract

**Background:**

Accumulated literature suggests that the acidic nuclear phosphoprotein 32 kilodalton (Anp32) proteins control multiple cellular activities through different molecular mechanisms. Like other Anp32 family members, Anp32e (a.k.a. Cpd1, PhapIII) has been conserved throughout vertebrate evolution, suggesting that it has an important function in organismal survival.

**Principal Findings:**

Here, we demonstrate that the Anp32e gene can be deleted in mice without any apparent effect on their wellbeing. No defects in thymocyte apoptosis in response to various stresses, fibroblast growth, gross behaviour, physical ability, or pathogenesis were defined. Furthermore, combined deletion of Anp32a and Anp32e also resulted in a viable and apparently healthy mouse.

**Significance:**

These results provide evidence that significant functional redundancy exists among Anp32 family members.

## Introduction

The acidic nuclear phosphoprotein 32 kilodalton (Anp32) proteins are metazoan-specific factors that have been implicated in the regulation of a panoply of cellular functions, including proliferation [1], [2], [3], [4], [5], apoptosis [6], [7], [8], [9], [10], [11], [12], [13], and differentiation [14], [15], [16], [17], [18]. Diverse Anp32 family members exert their functions by modulating phosphatase activity [19], [20], [21], mRNA stability [22], [23], [24], intracellular transport [22], [25], [26], caspase activation [8], [9], [10], [11], and/or chromatin modification [27], [28], [29]. Based on this accumulated literature, it is probable that the Anp32 factors control multiple cellular activities in a broad range of physiological contexts.

The mammalian Anp32 family is comprised of Anp32a (also known as LANP, pp32, I2PP2A, or PHAPIa), Anp32b (a.k.a. APRIL, PAL31, or PHAPIb), and Anp32e (a.k.a. Cpd1 or PhapIII) [30], [31]. All Anp32 proteins share two regions of amino acid conservation: the N-terminal leucine-rich repeat (LRR) domain and the C-terminal acidic tail. Although the Anp32 LRR domain resembles the protein-protein interaction domains of many other protein families, the Anp32 acidic tail is highly unusual in its amino acid composition [31]. The Anp32 acidic tail contains >100 amino acids, of which >80% are glutamate or aspartate residues. However, this sequence is not unique to the Anp32 family, as short conserved acidic regions resembling the Anp32 acidic tail can be found in the Anp32-binding partner the SET translocation protein [31].

Anp32e is the least well-characterized member of the mammalian Anp32 family. Anp32e was originally cloned as a gene upregulated in the mouse brain upon postnatal development, which led to suggestions that it might play a role in neuronal proliferation and differentiation [16]. Anp32e was also identified as having a potential oncogenic function in gastric cancer [32] and myeloma [33], but then was linked to favorable treatment outcomes in patients with follicular lymphoma [34]. Biochemical studies have indicated that Anp32e is a positive regulator of caspase activation in the apoptosome. Like other Anp32 family members, Anp32e can reduce the dATP required during apoptosome formation to conform with physiological levels of this nucleotide [9].

Despite the evolutionary conservation of Anp32 family members among metazoans, mutation of the Anp32 genes does not appear to affect embryogenesis. Flies bearing a validated mutation of mapmodulin, the Drosophila homologue of Anp32, are viable [35]. In mice, deletion of Anp32a results in no detectable health deficits or defects in neuronal function [36]. In humans, no mutations in any Anp32 family members have been linked to hereditary disorders. This lack of phenotype across multiple species has led to the hypothesis that the three mammalian Anp32 orthologues are functionally redundant such that no one factor is essential for development.

In this study, we confirm that the Anp32e gene can be deleted in mice without any apparent effect on their wellbeing. No defects in cellular growth, apoptosis, or neurological functions could be found. Furthermore, combined deletion of Anp32a and Anp32e also resulted in a viable and apparently healthy mouse. These results provide strong evidence that significant functional redundancy exists among Anp32 family members.

## Results

### Generation of Anp32e-deficient mice

**Figure 1:**
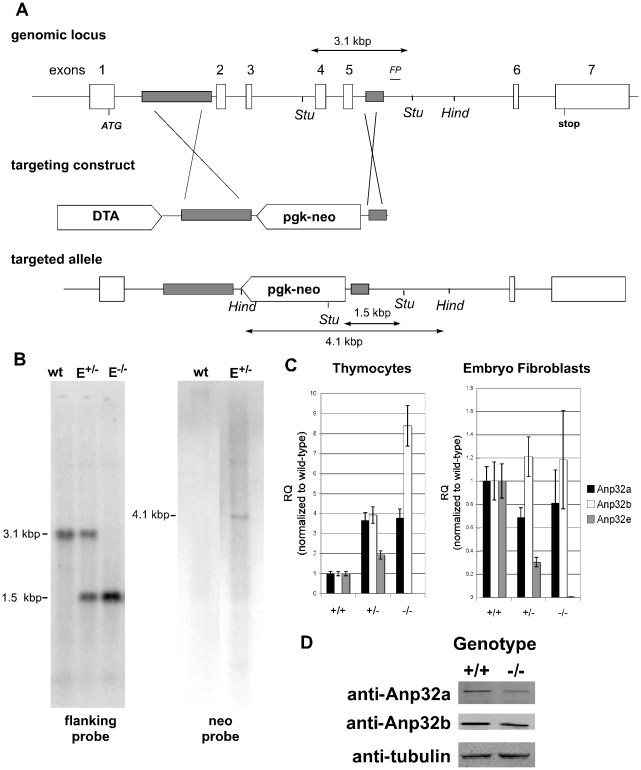
Generation and Validation of Anp32e-deficient mice. A. Targeting of the Anp32e gene. The diagram shows the murine genomic Anp32e locus and the targeting construct that replaced exons 2 to 6 with a pgk-neo cassette. Regions of homology in the targeting construct are shaded. *Stu,* StuI sites used for Southern blotting. *Hind*, HindIII sites used for Southern blotting of neo. *FP*, flanking probe. The sizes of the diagnostic Stu1 fragments for the wild-type Anp32e allele (3.1 kb) and the targeted Anp32e allele (1.5 kb) as well as the diagnostic HindIII fragment for neo insertion (4.1 kb) are shown. B. Confirmation of deletion. DNA from fetal Anp32e^+/+^ (wt), Anp32e^+/−^ (E^+/−^), and Anp32e^−/−^ (E^−/−^) mice was subjected to Southern blotting using the flanking probe in shown in diagram 1A (within intron 6). DNA from Anp32e^+/+^ (wt) and Anp32e^+/−^ (E^+/−^) ES cells was also subjected to southern blotting with a neo probe in order to confirm a single vector insertion. C. Validation of Anp32e mRNA deficiency. Quantitative RT-PCR of mRNA from primary MEFs from Anp32e^+/+^, Anp32e^+/−^ and Anp32e^−/−^ embryos at E14.5, and (bottom) thymocytes from Anp32e^+/+^, Anp32e^+/−^ and Anp32e^−/−^ mice at 4–8 weeks of age. Expression levels of Anp32a (black bars), Anp32b (grey bars), and Anp32e (white bars) mRNAs are shown relative to levels in Anp32e^+/+^ mice. Error bars represent the standard deviation from the mean across three technical replicates per sample. D. Absence of compensatory induction of other Anp32 proteins. Protein extracts from lymphocytes of Anp32e+/+ (wt) and Anp32e−/− (E−/−) mice were probed for Anp32a, Anp32b, and beta-tubulin expression.

To determine whether Anp32e had a unique biological role, we generated a targeting construct that resulted in the constitutive elimination of Anp32e exons 2–5 in one clone of murine ES cells (Figure 1A). Southern blotting of fetal mouse DNA using flanking probes confirmed the deletion of exons 2–5 (Figure 1B). Mutant mice were generated by standard blastocyst injection and backcrossed for six generations into the C57BL6 background. Quantitative RT-PCR analysis of mRNA from MEFs and thymocytes from Anp32e^+/+^ and Anp32e^−/−^ mice showed that no transcripts were expressed from the targeted Anp32e allele (Figure 1C). Using the same cDNA preparations to test for Anp32a and Anp32b mRNA expression, we found that these transcripts may be induced by deletion of Anp32e, particularly in thymocytes (Figure 1C). To more closely examine this potential induction, we tested whether protein levels of Anp32a and Anp32b were also increased in Anp32e^−/−^ thymocytes. As shown in Figure 1D, there was no evident compensatory induction of either Anp32a or Anp32b protein levels in the absence of Anp32e.

We attempted to generate monoclonal and polyclonal antisera specific for Anp32e to confirm the loss of Anp32e protein in our mutant mice. Unfortunately, none of the antisera tested produced a positive signal of the expected size when used in standard immunoblotting. Procurement of additional antisera and oligobodies courtesy of Dr. Martín Radrizzani [16] resulted in western blot bands of identical sizes in extracts of Anp32e^+/+^ and Anp32e^−/−^ MEFs and splenocytes (data not shown), suggesting that the protein detected here and in previous reports was not Anp32e.

### Anp32e-deficient mice are viable and fertile

A colony of Anp32e^+/−^ mice of mixed genetic background was established to determine the phenotypic impact of loss of Anp32e function. Interbreeding of Anp32e^+/−^ mice produced Anp32e^−/−^ mice at the expected Mendelian ratio, at least at time of weaning (3–4 weeks of age; Table 1). When mice in this colony were examined for fertility, we found that both male and female Anp32e^−/−^ mice of 6–40 weeks of age were able to produce normal-sized litters at normal frequencies when bred to their Anp32^+/−^ counterparts (data not shown). These findings suggest that loss of Anp32e function does not deleteriously affect the development or fertility of mice under normal laboratory conditions.

**Table 1:** Expected and attained Mendelian ratios of progeny derived from the intercrossing of Anp32e^+/−^ mice.

	genotype		
	Anp32e^+/+^	Anp32e^+/−^	Anp32e^−/−^
expected	8.5 (25%)	17 (50%)	8.5 (25%)
attained	10 (29.4%)	16 (47.0%)	8 (23.5%)

Data shown are the number of mice of a given genotype (percentage of total progeny). Chi square analysis determined that there was no significant difference from expected ratios.

### Anp32e^−/−^ cells show normal rates of proliferation and apoptosis

**Figure 2:**
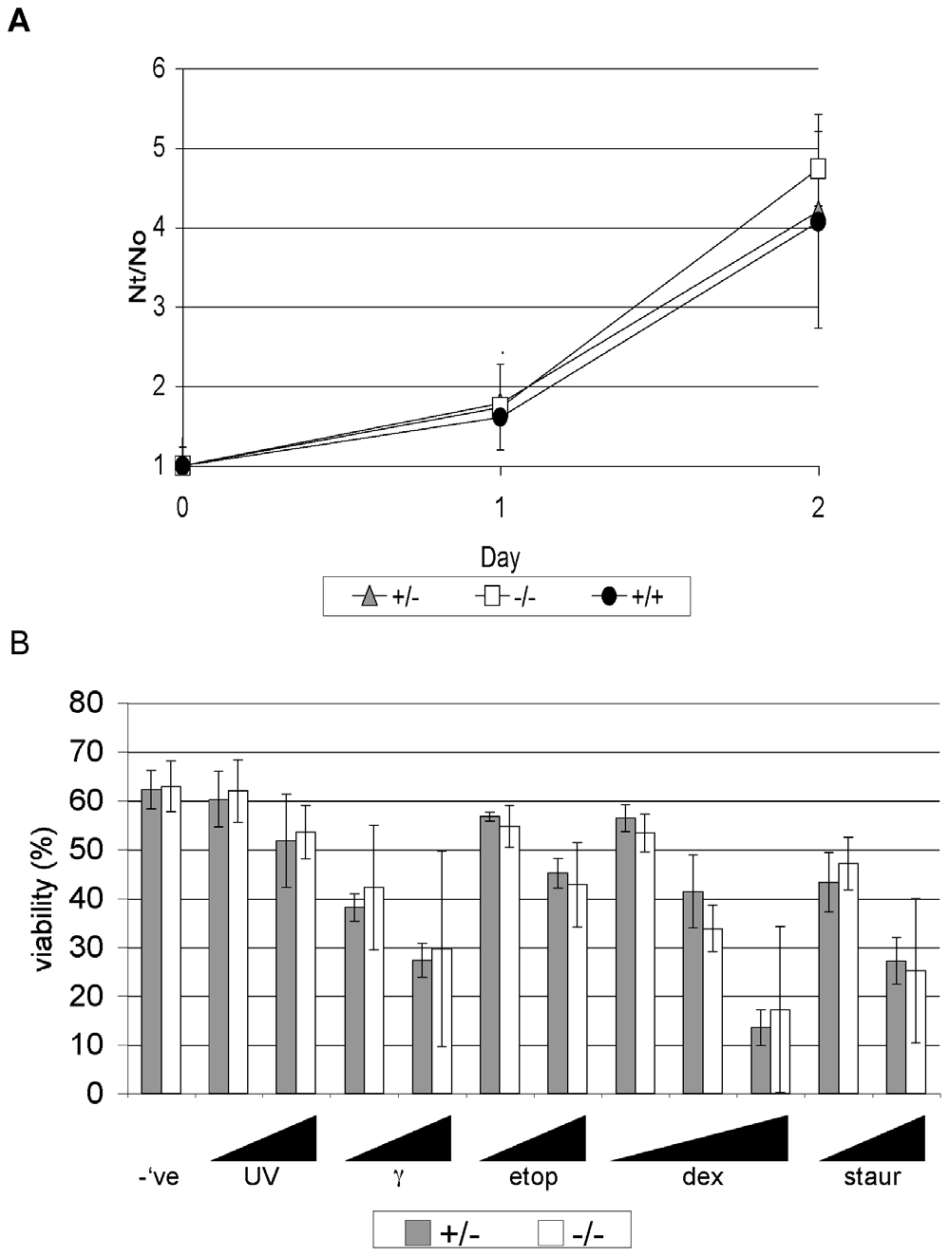
Normal proliferation and apoptosis of Anp32e^−/−^ cells. A. Growth curves of primary MEFs from Anp32e^+/+^, Anp32e^+/−^ and Anp32e^−/−^ mice (n = 2/genotype). *Nt/N0*, cell number at time point/cell number on day 0. B. Apoptosis of Anp32e^+/−^ and Anp32e^−/−^ thymocytes after 20 hours in culture response to (left to right): –ve, untreated controls, UV-irradiation (30 mJ/cm^2^ and 60mJ/cm^2^), γ-irradiation (1 Gy and 2 Gy), etoposide (1 µM and 3 µM), dexamethasone (3 nM and 10 nM), and staurosporine (1 µM and 3 µM). Results shown are mean % viable cells ± SD (n = 3/genotype).

Because the Anp32 proteins have been linked to cell proliferation**,** we examined whether there was any difference in proliferation rates between Anp3e^+/+^ and Anp32e^−/−^ cells. We isolated primary MEFs from Anp32e^+/+^, Anp32e^+/−^ and Anp32e^−/−^ mice from a single litter of C57/BL6 congenic animals and examined their growth *in vitro.* No differences were detected in a two-day analysis (Figure 2A). Furthermore, an absence of Anp32e did not alter the kinetics of release of MEFs from serum starvation-induced arrest (data not shown). These data suggest that Anp32e does not play a unique role in proliferation under either normal or low serum conditions.

Anp32e has been identified as an activator of apoptosome-mediated proteolysis [9]. To test whether Anp32e-deficient cells were defective in intrinsic apoptotic signaling, we isolated thymocytes from Anp32e^+/+^ and Anp32e^−/−^ mice and exposed them to various apoptotic stimuli. As shown in Figure 2B, these thymocytes responded in equivalent fashion to both intrinsic and extrinsic apoptotic stimuli, suggesting that Anp32e does not play a unique role in apoptosis.

### Anp32e-deficient mice demonstrate no apparent neurological defects

**Figure 3:**
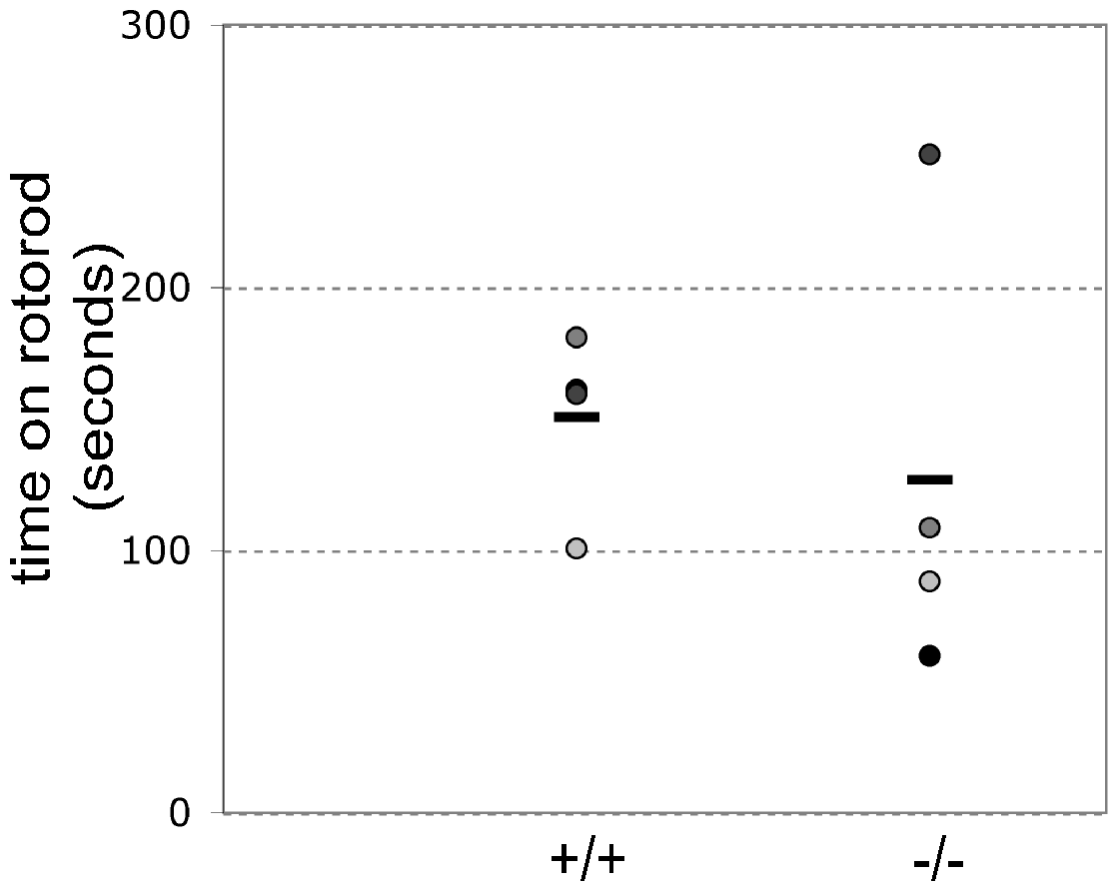
Normal rotorod behaviour. Individual Anp32e^+/+^ and Anp32e^−/−^ mice (spherical symbols) were placed on a rotating rotorod and the time they remained suspended was measured in seconds. Horizontal bars, mean duration per genotype.

It has been suggested that Anp32 proteins might play roles in brain development [16], [37]. To test this hypothesis in our mutant mice, we first performed gross histological examinations of the brains of C57BL/6-congenic Anp32e^−/−^ and Anp32e^+/+^ mice. We found no structural differences between wild-type and mutant brains, and the sizes and cellularity of various regions of these brains were comparable (data not shown). We then tested Anp32e^−/−^ and Anp32e^+/+^ mice for stimulatory responses in qualitative examinations and found that hearing and whisker stimulation evoked similar responses in both strains. Finally, we examined the relative abilities of these animals to perform in a timed Rotarod suspension assay. There was no statistically significant difference between the mean time that Anp32e^−/−^ mice were able to remain suspended (126 sec) and the mean suspension time of Anp32e^+/+^ littermates (151 sec; Figure 3). Thus, a lack of Anp32e does not impair balance and motor control in mice.

### Anp32e-deficient mice demonstrate no specific pathologies

Because our Anp32e-deficient mice showed no apparent deficits in any of the functions proposed for Anp32e, we expanded our phenotypic analyses and used standard assays of blood chemistry and hematology to perform a broad-spectrum analysis of internal organ function. No statistically significant differences between sex-matched Anp32e^+/+^ and Anp32e^−/−^ littermates at 4–8 weeks of age were detected in clinical chemistry (Table 2, top), suggesting that the functions of the liver [as defined by albumin, alanine transaminase (ALT), and aspartate transaminase (AST) levels], the heart [as defined by creatine kinase (CK) and lactate dehydrogenase (LDH) levels], the pancreas [as defined by amylase and glucose levels], and the kidney [as defined by creatinine, uric acid, and ion levels] are not significantly affected by loss of Anp32e. Furthermore, gross hematological examination demonstrated no differences in leukocyte or erythrocyte counts (Table 2, bottom), suggesting that Anp32e deficiency has no effect on basic blood constituents. When heart function was examined using ECG, the results obtained for Anp32e^−/−^ mice were within normal variability (data not shown).

**Table 2:** Clinical chemistry and hematology comparisons of 4 pairs of Anp32e^+/−^ and Anp32e^−/−^ littermate mice.

		Anp32e^+/+^	Anp32e^−/−^
Clinical chemistry	Albumin (g/L)	35.3±1.7	35.5±1.7
	ALT (Units/L)	25.8±6.0	31.6±3.6
	AST (Units/L)	50.8±4.3	53.0±5.6
	CK (Units/L)	192.5±92.4	373.8±211.7
	LDH (Units/L)	397.3±48.9	466.0±80.3
	Amylase (Units/L)	2478.8±358.3	2604.5±515.6
	Cholesterol (mM)	3.0±0.6	2.9±0.7
	Triglycerides (mM)	0.7±0.1	0.7±0.2
	Glucose (mM)	12.9±1.1	11.1±1.3
	Creatinine (c)	16.3±1.5	11.3±5.1
	Uric Acid (µM)	65.8±18.6	61.5±19.2
	Na^+^ (mM)	147.0±1.8	145.0±2.0
	K^+^ (mM)	5.4±0.3	5.5±0.6
	Cl^-^ (mM)	107.5±1.7	107.0±0.0
Hematology	RBC (billions/mL)	11.1±0.5	11.1±0.3
	HgB (g/L)	166.8±7.8	161.5±6.2
	HCT (L/L)	0.49±0.02	0.48±0.02
	MCV (fL)	43.7±0.3	43.0±0.9
	MCH (pg/cell)	15.0±0.2	14.6±0.3
	MCHC (g/L)	342.3±3.0	340.0±3.4
	PLT (millions/mL)	1146±200	1221±70
	WBC (millions/mL)	15.6±0.9	15.3±0.7

Data shown are the mean levels of the indicated parameters ± SD (n = 4/genotype). *ALT* , Alanine transaminase. *AST*, Aspartate transaminase. *CK*, Creatine kinase. *LDH*, Lactose dehydrogenase. *Na^+^*, Sodium. *K^+^*, Potassium. *Cl^-^*, Chloride. *HgB*, Hemoglobin. *HCT*, Hematocrit. *MCV*, Mean corpuscular volume. *MCH*, Mean corpuscular hemoglobin. *MCHC*, Mean corpuscular hemoglobin concentration. *PLT*, Platelet count. *WBC*, White blood cell count.

To search for a function of Anp32e in preventing disease, we allowed Anp32e^+/−^ and Anp32e^−/−^ littermate mice of a mixed background to age and monitored them for susceptibility to early disease onset. We found that 5/6 Anp32e^−/−^ mice were still alive at 80 weeks of age when the experiment was terminated; this proportion was similar to that in the Anp32e^+/−^ group (data not shown). None of the mutants showed any gross defects or tissue pathologies. These results indicate that either the function of Anp32e is redundant with that of another molecule, or that its unique function will only be revealed by the application of an unusual stress yet to be determined.

### Double Anp32a;Anp32e deficiency does not affect viability

**Figure 4:**
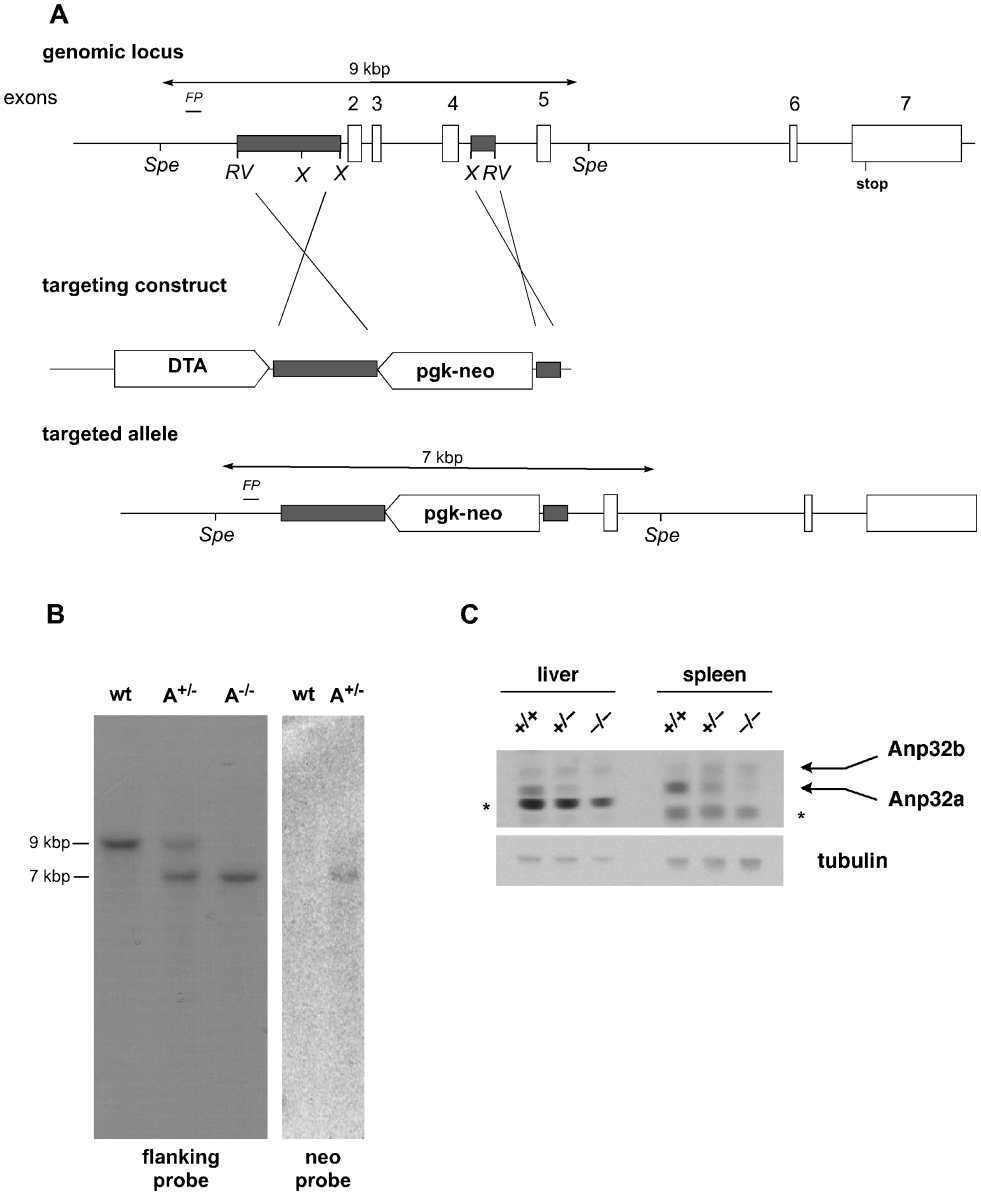
Generation and Validation of Anp32a-deficient mice. A. Targeting of the Anp32a gene. The diagram shows the murine genomic Anp32a locus and the targeting construct that replaced exons 2 to 5 with a pgk-neo cassette. Regions of homology in the targeting construct are shaded. *RV,* EcoRV; X, XbaI; Spe, SpeI; *FP*, flanking probe used for Southern blot analysis. B. Confirmation of deletion. DNA from embryos of Anp32a^+/+^ (wt), Anp32a^+/−^ (A^+/−^) and Anp32a^−/−^ (A^−/−^) was subjected to Southern blotting using the flanking probe in A (within intron 1). Anp32a^+/+^(wt) and Anp32a^+/−^ (A^+/−^) ES-cell DNA was also probed for the neomycin resistance gene (Neo probe). C. Validation of Anp32a protein deficiency by immunoblotting of extracts from mouse liver and spleen. Primary anti-APRIL antibody raised in mice against recombinant human Anp32b protein recognizes both the murine Anp32b protein and the Anp32a protein, which can be distinguished by size. Tubulin, loading control. *, non-specific bands seen in liver and spleen.

**Figure 5:**
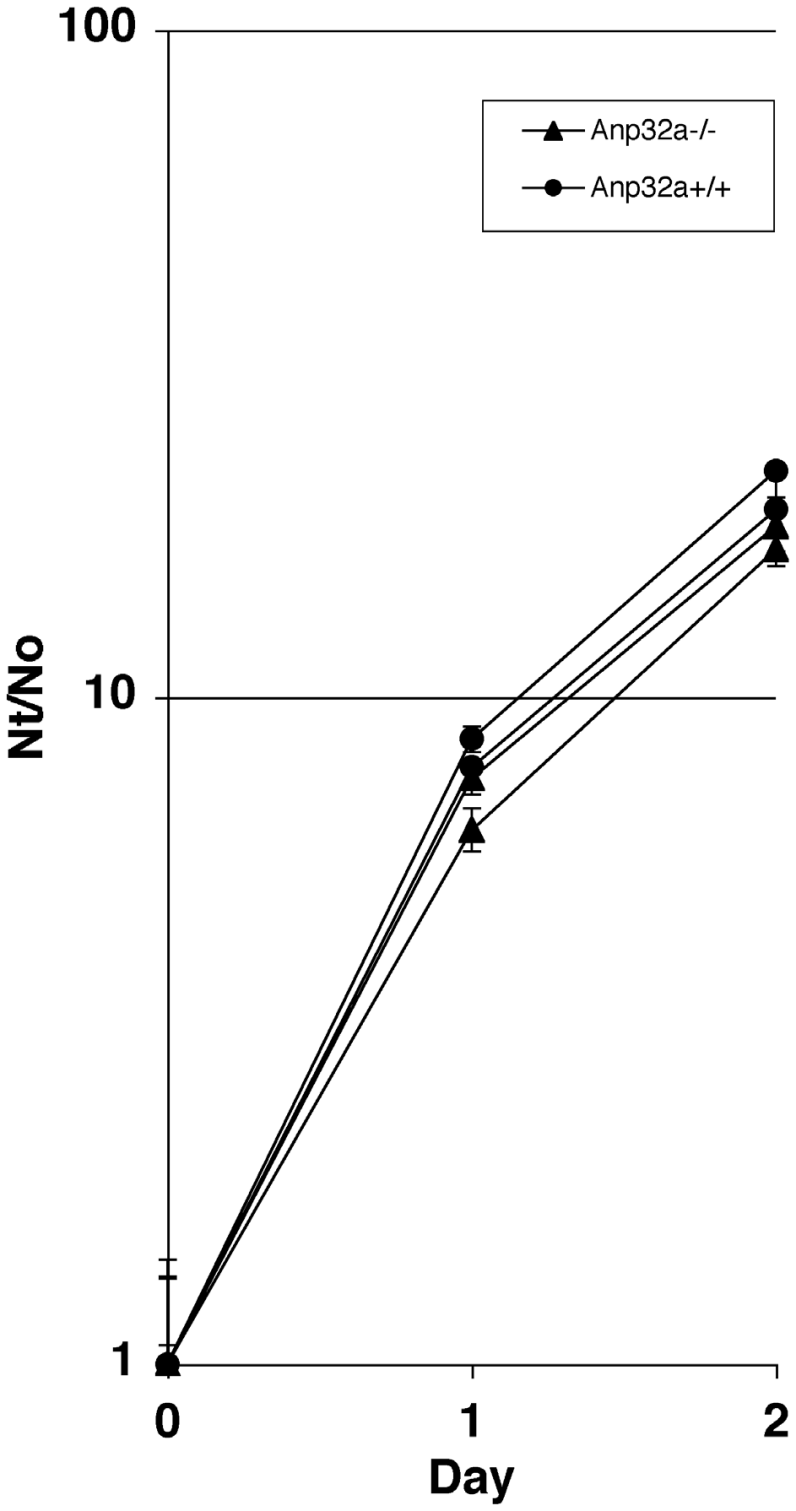
Growth Curve of Anp32a-deficient fibroblasts. Growth curves of early passage primary MEFs from littermate isolates of Anp32a^+/+^ and Anp32a^−/−^ embryos. Two isolates per genotype to give four littermate MEF lines total. *Nt/N0*, cell number at time point/cell number on day 0.

In a further attempt to delineate Anp32e's physiological role, we decided to create double mutant Anp32e^−/−^;Anp32a^−/−^ mice. First, we generated a novel strain of Anp32a^−/−^ mice (Figure 4). Deletion of the genomic sequence was confirmed by Southern blotting using a flanking probe as well as a neomycin probe (Figure 4B). Anp32a protein deletion was confirmed by immunoblot analysis of liver and spleen extracts using a polyclonal anti-APRIL antibody [23] that crossreacts with Anp32a (Figure 4C). Consistent with a previous report [36], we found no difference in viability of the Anp32a mutation in either a mixed or backcrossed C57BL6 background (data not shown). To examine potential physiological changes in Anp32a-deficient cells, we performed growth curve analysis on early-passage mixed-bred Anp32a−/− embryo fibroblasts (Figure 5). We found no significant difference in net proliferation in this analysis.

We then bred our Anp32e^+/−^ animals to our Anp32a^+/−^ mice to generate double mutant progeny of a mixed 129Ola:C57BL6 background, and intercrossed the double heterozygotes to examine all potential genotypes. We found that the Anp32e^−/−^ genotype was present at the expected Mendelian ratio in both in the Anp32a^+/−^ and Anp32a^−/−^ backgrounds (Table 3). In fact, no statistically significant deviations from expected Mendelian ratios were observed for this breeding in its entirety. Investigation of a small number of litters showed that both males and females of the Anp32a^−/−^;Anp32e^−/−^ genotype were fertile and able to produce litters of normal size and gross phenotype.

**Table 3:** Expected and attained Mendelian ratios of progeny derived from the intercrossing of Anp32e^+/−^;Anp32a^+/−^ double mutant littermate mice.

	Genotype								
	Anp32a^+/+^;Anp32e^+/+^	Anp32a^+/+^;Anp32e^+/−^	Anp32a^+/+^;Anp32e^−/−^	Anp32a^+/−^; Anp32e^+/+^	Anp32a^+/−^; Anp32e^+/−^	Anp32a^+/−^; Anp32e^−/−^	Anp32a^−/−^; Anp32e^+/+^	Anp32a^−/−^; Anp32e^+/−^	Anp32a^−/−^; Anp32e^−/−^
Expected	2 (7.4%)	3 (11.1%)	2 (7.4%)	3 (11.1%)	7 (25.9%)	3 (11.1%)	2 (7.4%)	3 (11.1%)	2 (7.4%)
Attained	3 (11.1%)	6 (22.2%)	3 (11.1%)	3 (11.1%)	3 (11.1%)	6 (22.2%)	1 (3.7%)	0 (0%)	2 (7.4%)

Data shown are the number of mice of a given genotype (percentage of total progeny). Chi square analysis determined that there was no significant difference from expected ratios.

## Discussion

Like other Anp32 family members, Anp32e has been conserved throughout vertebrate evolution, suggesting that it has an important function in organismal survival. However, our study demonstrates that ablation of Anp32e in mice does not affect their development or general health. Even the application of apoptotic stresses, behavioural tests, and aging experiments failed to elicit a phenotype for Anp32e-deficient animals. Although we do not claim that our analyses were exhaustive, we have performed a broad range of experiments designed to reveal effects on all currently reported functions of Anp32e.

Our work has raised an issue with respect to interpreting prior studies on Anp32e, particularly those based on the use of antibodies and oligobodies. Our investigation showed that tissues from a mutant mouse whose Anp32e deficiency was validated by quantitative RT-PCR produced an identical band pattern to that of wild-type tissues upon western blotting. Such a result suggests that the antibody and oligobody used in previous publications [16], [19] may not recognize Anp32e at all but rather an unknown factor(s).

Anp32e is the second Anp32 family member whose deletion in mice results in no apparent phenotype. Two independent loss-of-function alleles of Anp32a have now been tested in mice and have yielded no phenotype [36]. These findings all point to a probable functional redundancy of Anp32 genes, consistent with the conservation of specific protein domains among family members.

Although functional redundancy with other Anp32 family members is one possible explanation for the survival of Anp32 single mutants, the conservation of all three factors across species suggests that these genes do indeed have individual independent functions. The *in vivo* function(s), as elucidated from knockout analyses, may only be activated in response to stresses that remain to be identified. Alternatively, it may be that a complete functional overlap of the Anp32 genes exists to ensure that potentially catastrophic developmental errors are overcome.

## Materials and Methods

### Mice

Mice were maintained under SPF conditions in individually ventilated cages and fed 5% irradiated meal. Unless otherwise noted, all Anp32^−/−^ mice analyzed in this study were derived from a single clone of embryonic stem (ES) cells and backcrossed six generations into the congenic C57/BL6 background.

### Plasmids and primers

Sequences directing the expression of Diphtheria toxin A in mouse ES cells were cloned into pBluescript (pgk-neo) [38] to give the plasmid pBSneoDTA. Regions of the Anp32e gene adjacent to the targeted exons were cloned by high fidelity PCR. The primers GGGGTCGACCGTGAAATCGAGGGACAGGGGTCCGC and GGGGGATCCCAGACCCTTCGGTGTCTAAGCATTCTAAACGCATG were used to clone the upstream arm of homology between the SalI and BamHI sites. The primers GGGCCGCGTAAACTGGCCTTGAGGTGAGACCAGTGG and GGGGCGGCCGCGAGTGCTGGGATTAAAGGCGTGCGCCACC were used to clone the downstream arm of homology between the XbaI and NotI sites. Homologous regions of Anp32a adjacent to the targeted exons were cloned as the EcoRV-XbaI fragment upstream of exon 2 and the XbaI-EcoRV fragment downstream of exon 4.

### Gene targeting

Targeting constructs were linearized and transfected by electroporation into E14K mouse ES cells as previously described [39]. Homologous recombinants were selected by culture in 0.3 mg/ml G418 for 10–14 days and screened for appropriate integration by PCR and southern blotting as previously described [38], [39].

### Southern blotting

Southern probes used to detect Anp32 genomic sequences were amplified from genomic ES DNA. Primer pair sequences were as follows: Anp32e, CCTGTGAATGTGTGGCGTGTGAATGTAG and TTTCCCACTCAATGCACTGGCTCTTAGA; Anp32a, CCATGGATTCTGTGACCCTTCAGCC and CCCAAGTCACCTACCATTGCACCC. Genomic DNA was digested, resolved on gels, transferred to nylon membranes, and hybridized to the above probes using standard procedures [38], [39]. To optimize image sizing, some figures have been adjusted so that desired lanes appear beside one another.

### Quantitative RT-PCR

RNA from adult mouse tissues was isolated using a commercial kit and protocol (Qiagen). cDNA was reverse-transcribed from 1 µg mRNA using Superscript II enzyme (Invitrogen), and analyzed by Syber-Green nucleotide incorporation in separate PCR reactions. Primers were:

Gapdh (loading control), AACAGGAAGCCCATCACATCTT and GCCCTTCCACAATGCCAAAGTT; Anp32a, GCGAAAACAGAATCTCAGGGG and TTCTTCAGCGGCTCTATTGTG; Anp32b, AGCCGTTCGAGAACTTGTCTT and CAGGTTATTGCCACTTAGGTTCA; Anp32e, AATTGCTTGTGTGTCAATGGGG and GGCCATGCTAAGAAACTCCAG.

### Western blotting

Immunoblotting to detect Anp32a was performed on 5 µg protein extracted by NP-40 detergent lysis of homogenized mouse thymuses, livers, or spleens. After SDS-PAGE resolution, proteins were transferred to PDVF membranes and probed with anti-β-tubulin (loading control; Li-Cor Bioscience), anti-APRIL antibody that detected both the Anp32a and Anp32b proteins (courtesy of Dr. Imed Gallouzi, McGill University, Montreal, Canada) [23], anti-Anp32a (sc-5652, Santa Cruz Biotech), or anti-Anp32b (10843-1-AP, Proteintech). Bands on blots were visualized according to Li-Cor Odyssey standard protocols.

### Rotorod analysis

Four littermate pairs of Anp32e^+/+^ and Anp32e^−/−^ male mice (4–8 weeks old) were examined for their ability to stay on the Economex Rota Rod when the apparatus was operating at an initial speed of 4 rpm that subsequently increased at a rate of 0.1 rpm/second. Each mouse was tested three times with a 15 min rest period between tests.

### Clinical chemistry, hematology and electrocardiography

Blood was extracted from the saphenous vein of 4 pairs of Anp32e^+/+^ and Anp32e^−/−^ male littermates at 4–8 weeks of age. Samples were sent to IDEXX Laboratories Inc. for commercial analysis of wide spectrum clinical chemistry. Additional blood samples from the same mice were tested for hematological differences using a Coulter A^c^•T diff Analyzer (Coulter Corp.). Electrocardiograms (ECG) were generated for these same mice using the Power Lab/4SP system with ML135 and MLA0112 attachments (AD Instruments). The 5 electrodes of this apparatus were connected subcutaneously at the limbs. The ECG were analyzed using the SAECG extension for Chart 4 (AD Instruments).

### Apoptosis

Apoptosis assays were performed as described previously [39], [40]. Briefly, single cell suspensions were prepared of total thymocytes from three sex-matched littermate pairs of Anp32e^+/+^ and Anp32e^−/−^ mice (4–8 weeks old). The cells were exposed to apoptotic stimuli as detailed in Figure 2B and then cultured overnight in RPMI medium containing penicillin/streptomycin, 10% fetal bovine serum (FBS) and L-glutamine. Viability was determined by propidium iodide [16] exclusion and flow cytometry using a FACScaliber (BD Pharmingen).

### Growth curves

Growth curves were established as described previously [41]. Briefly, primary murine embryonic fibroblasts (MEFs) were derived from 2 embryos/genotype (Anp32e^+/+^, Anp32e^+/−^, Anp32e^−/−^) on embryonic day 14.5 (E14.5). MEFs were cultured in Dulbecco’s MEM containing penicillin/streptomycin, 10% FBS, L-glutamine, and beta-mercaptoethanol. After two passages, the cells were plated at 5×10^4^ cells/well in 6-well dishes. For serum deprivation studies, the medium was replaced the next day (Day 0) with medium containing 0.1% FBS. For quantitation, triplicate wells for each genotype and each time point (day 0, 1 or 2) were washed in PBS and trypsinized. Cells were collected in specified volumes and counted on a hemocytometer. To eliminate small variations in seeding and plating efficiency, growth was normalized to the initial cell count on Day 0.

### Ethics Statement

All animal treatments were approved in advance by the University Health Network Animal Care Committee, protocol numbers 0985 and 02147, and performed in the Ontario Cancer Institute Animal Research Centre, Ontario registry number 0050.
